# Extracorporeal delivery of a therapeutic enzyme

**DOI:** 10.1038/srep30888

**Published:** 2016-08-01

**Authors:** Chun Zhang, Jun Pu, Xiaolan Yang, Tao Feng, Fang Liu, Deqiang Wang, Xiaolei Hu, Ang Gao, Hongbo Liu, Chang-Guo Zhan, Fei Liao

**Affiliations:** 1 Key Laboratory of Medical Laboratory Diagnostics of the Education Ministry, College of Laboratory Medicine, Chongqing Medical University, No. 1, Yixueyuan Road, Chongqing 400016, China; 2 Department of Urology, the First Affiliated Hospital of Chongqing Medical University, No. 1, Youyi Road, Chongqing 400016, China; 3 Molecular Modeling and Biopharmaceutical Center and Department of Pharmaceutical Sciences, College of Pharmacy, University of Kentucky, 789 South Limestone Street, Lexington, KY 40536, USA

## Abstract

To remove circulating harmful small biochemical(s)/substrates causing/deteriorating certain chronic disease, therapeutic enzyme(s) delivered *via* vein injection/infusion suffer(s) from immunoresponse after repeated administration at proper intervals for a long time and short half-lives since delivery. Accordingly, a novel, generally-applicable extracorporeal delivery of a therapeutic enzyme is proposed, by refitting a conventional hemodialysis device bearing a dialyzer, two pumps and connecting tubes, to build a routine extracorporeal blood circuit but a minimal dialysate circuit closed to circulate the therapeutic enzyme in dialysate. A special quantitative index was derived to reflect pharmacological action and thus pharmacodynamics of the delivered enzyme. With hyperuricemic blood *in vitro* and hyperuricemic geese, a native uricase *via* extracorporeal delivery was active in the dialysate for periods much longer than that *in vivo* through vein injection, and exhibited the expected pharmacodynamics to remove uric acid in hyperuricemic blood *in vitro* and multiple forms of uric acid in hyperuricemic geese. Therefore, the extracorporeal delivery approach of therapeutic enzymes was effective to remove unwanted circulating small biochemical(s)/substrates, and was expected to avoid immunogenicity problems of therapeutic enzymes after repeated administration at proper intervals for a long time due to no contacts with macromolecules and cells in the body.

Certain chronic disease, such as chronic kidney failure or metabolic deficiency/disorder, is caused and/or deteriorated by harmful small biochemical(s) in blood. The treatment of such a chronic disease requires removal of the circulating harmful small biochemical(s), mainly by repeated administration of hemodialysis or therapeutic enzyme(s)^1^,^2^,^3^,^4^. However, hemodialysis is limited by dialysis disequilibrium syndrome and the imbalance of small nutrients/hormones. Therapeutic enzymes may be delivered *via* vein infusion or injection, but suffer from short circulation half-lives since delivery and immunogenicity after repeated administration at proper intervals for a long time. For example, gout as a chronic disease is caused and deteriorated by blood uric acid at elevated levels; a therapy to reduce blood uric acid levels is required for gout treatment^5^,^6^. Specially, refractory gout is a major medical challenge since conventional chemical agents are ineffective or intolerable, and enzyme therapy with uricase is the only effective treatment to date^7^,^8^,^9^,^10^. Currently, pegloticase (a formulated uricase) *via* vein infusion is initially effective in treatment of refractory gout, but its repeated use biweekly over three months ultimately elicits immunoresponse in most patients to greatly shorten its half-life in blood since delivery, and thus mitigate its therapeutic safety and efficacy^8^,^9^,^10^. To solve the immunogenicity problem, there must be an engineered/formulated enzyme of negligible immunogenicity, or the enzyme should be delivered *via* a novel approach that can avoid the immunoresponse of patients after repeated administration at proper intervals for a long time. Molecular engineering and/or formulation of a therapeutic enzyme to solve its immunogenicity problem remains to be a grand challenge. To employ an enzyme for repeated treatment of a chronic disease at proper intervals over a long time, it is thus highly desired to develop a novel delivery approach that can avoid the immunogenicity problem.

Conventional delivery of a therapeutic enzyme includes vein infusion, or injection through vein, muscle or skin, and ultimately leads to the therapeutic enzyme circulating in blood. Generally speaking, of a therapeutic enzyme through the conventional delivery, the immunogenicity after repeated administration at proper intervals for a long time and short circulation half-life since every delivery are the two principal problems, and caused primarily by the interactions of the therapeutic enzyme with cells and proteins in the body. When a small substrate is separated from cells and proteins in blood using a medical device and only the isolated small substrate is accessible to a therapeutic enzyme in the device, the blood cells and proteins in the body will not contact the therapeutic enzyme and, therefore, there will be no immunoresponse at all even after repeated administration of the enzyme at proper intervals for a long time. For the required separation, a conventional hemodialysis system can be employed technically, whose basic configuration includes a dialyzer, two pumps and connecting tubes to form an extracorporeal blood circuit and a dialysate circuit. When the dialysate circuit is closed to circulate a small volume of dialysis solution containing the therapeutic enzyme, the modified hemodialysis system becomes the device for the desired extracorporeal delivery of the therapeutic enzyme (which is uricase in this study) (Fig. 1). Putatively, hemodialysis system can be repeatedly administrated at proper intervals to patients for years. A native uricase, which is known to elicit strong immunoresponse when repeatedly administrated at proper intervals for a long time *via* conventional delivery, is thus expected to be applicable through extracorporeal delivery for the continued treatment of refractory gout at no cost of immunoresponse. Moreover, since every extracorporeal delivery, a therapeutic enzyme in the delivery device is expected to be active for a long period determined only by its stability in the dialysate because there is no elimination of the therapeutic enzyme by any factors from the delivery device at all. Clearly, the pharmacological efficacy of a therapeutic enzyme *via* extracorporeal delivery is the first of all. Herein, the pharmacological efficacy of the extracorporeal delivery approach was investigated and proved with a native uricase to remove uric acid in hyperuricemic blood *in vitro* and hyperuricemic geese.

## Results

### An extracorporeal delivery device and the experimental system

In this proof-of-principle study, the pharmacological action of a native uricase *via* the extracorporeal delivery to remove blood uric acid was tested by using hyperuricemia models *in vitro* and *in vivo*. The proposed extracorporeal delivery device employed licensed DTB-100 multiple-function pumps of Chongqing Duotai Medical Equipment Co. Ltd. (China, http://www.cqduotai.com/showimg.asp?id=6&foid=10), licensed UT-300S dialyzer from Nippro Medical Equipment Co. Ltd (Shanghai, China; http://www.nipro-trading.com.cn/), and dedicated sterile connecting tubes from Jiangxi Sanxin Medical Equipment Co. Ltd. (Jiangxi; http://www.sanxin-med.com/). To facilitate sampling, injection interfaces made from latexes were inserted at 2.0 cm before and after the dialyzer in the connecting tubes of the blood circuit, and at somewhere of the dialysate circuit. The volume of blood in the dialyzer and the connecting tubes reached 28 ml, which affected the selection of hyperuricemia model *in vivo*.

A recombinant bacterial uricase was prepared and its native form was directly used^11^,^12^. A hyperuricemia model *in vivo* can be developed with a potent inhibitor against the endogenous uricase of a laboratory animal. However, there are no steady levels of blood uric acid for a long time with such a hyperuricemia model, due to the metabolism of both the inhibitor and uric acid; this fact inevitably requires more experimental efforts to characterize the pharmacological action of the delivered uricase. Additionally, any inhibitor in blood will diffuse into the dialysate to interfere with the action of the delivered uricase, and thus complicate the pharmacodynamics of the delivered uricase. Fortunately, some avian animals lack uricase and have inherent hyperuricemia^13^. The volume of blood in the dialyzer and the connecting tubes indicated the need of a hyperuricemic animal model bearing total blood capacity over 300 ml since less than 10% of the total amount of blood in the body can be circulated extracorporeally. The total amount of blood (in liter) of an animal is usually assumed to be 6% of its body weight (in kg). Grown-up goose of body weight over 5.0 kg was thus the suitable hyperuricemia model *in vivo* for favorable cost^13^. Fresh goose blood sample of 200 ml was utilized as the hyperuricemia model *in vitro*. The extracorporeal circulation speed of blood was fixed at 15 ml/min, due to the limited tolerability of goose.

### Quantitative characterization of the pharmacological action of the delivered uricase

For extracorporeal delivery of anyone enzyme to remove its diffusible small substrate in blood *in vitro* or *in vivo*, the dynamic change of the total quantity of the small substrate in the experimental system reflects the pharmacodynamics. Consequently, the quantity of the small substrate eliminated from the experimental system within a short sampling interval during the continued action of the enzyme delivered in the device (SQ_i_) reflects the instantaneous pharmacological action; the sum of SQ_i_ over the continued action periods started from the same initial sampling timepoint indexes pharmacodynamics for enzymatic removal of the small substrate in blood. Clearly, transmembrane diffusion of the small substrate from blood into the dialysate is the prerequisite for its removal by the delivered enzyme. To simplify pharmacodynamics, the enzyme was added into the closed dialysate circuit after the equilibrium of its small substrate in the two circuits, and the device plus the enzyme was continuously running in a reasonable period for consistent permeability of the dialyzer. To facilitate dissolution of any small biochemicals in the dialysate, bovine serum albumin was added to the dialysate for a concentration consistent with that in plasma^14^. Moreover, there may be the deposit of the small substrate in the dialysate; the quantity of the small substrate deposited in the dialysate over a given period was calculated as the capacity of the dialysate circuit times the change of the levels of the small substrate in the dialysate within the action period. Furthermore, a small substrate may be deposited in non-blood tissues and transported into blood when its level in blood is significantly reduced. To quantitatively characterize the pharmacological action of the delivered enzyme after a given continued action period, SQ1_i_ and SQ2_i_ were thus utilized for applicability to different situations and estimated as follows.

SQ1_i_ considered the distribution of the small substrate in different tissues and the transportation of the small substrate from non-blood tissues into blood, which was discussed in detail with uric acid in goose as the example. Putatively, only free molecules of uric acid can diffuse across the dialyzer. However, free molecules, precipitates, soluble and insoluble complexes of uric acid are found in goose tissues^13^, and have different rates to be transported into the dialysate. Based on transportation kinetics, uric acid related to uricase action in the device was classified into the fast-diffusing and slow-diffusing forms. The fast-diffusing uric acid included free molecules and soluble complexes that exist only in the dialysate (compartment A) and the plasma (compartment B); there was rapid equilibrium among fast-diffusing uric acid in such two compartments when both blood and dialysate were circulating at proper speeds and the dialyzer membrane had sufficient permeability to uric acid. The slow-diffusing uric acid included precipitates and insoluble complexes that may exist in any tissues including blood itself, plus free molecules and soluble complexes inside cells; such localizations of slow-diffusing uric acid were assigned to compartment C and the transportation of uric acid from compartment C into dialysate required a much longer time. Clearly, the pharmacological action of the delivered uricase will remove uric acid in those three compartments through transportation of uric acid among tissues. However, the quantitative processing of such transportation of uric acid among tissues was quite challenging. Fortunately, it is the diffusion of uric acid from plasma into the dialysate that reduces uric acid concentration in blood right after the dialyzer against that right before the dialyzer. To reflect pharmacodynamics after a given continued action period of the delivered uricase with the same initial sampling timepoint, SQ1_i_ was estimated by numerical integration of the dynamic quantity of uric acid transported from plasma into the dialysate over the action period, subtracting the quantity of uric acid deposited in the dialysate within the same period. SQ1_i_ utilized just the physical principle of dialysis and was thus generally applicable regardless of the transportation of the small substrate among tissues.

SQ2_i_ considered no transportation of the small substrate from non-blood tissues into blood. SQ2_i_ was calculated as the total blood capacity times the reduction of the level of the small substrate in blood right before the dialyzer against its initial value, subtracting the quantity of uric acid deposited in the dialysate within the same action period (Supplementary Note S1). To reflect pharmacodynamics of the delivered enzyme, clearly, the calculation of SQ2_i_ was much simpler than that of SQ1_i_, but SQ2_i_ was limited to anyone small substrate in blood as the target exhibiting no transportation among tissues.

In practice, when three series of the concentrations of fast-diffusing uric acid, in blood right before and after the dialyzer, and in the dialysate, were determined with a special sampling order, SQ1_i_ and SQ2_i_ were accessible concomitantly (Supplementary Note S1). Notably, SQ1_i_ was valid but SQ2_i_ was invalid to reflect pharmacodynamics for the removal of blood uric acid by the action of the delivered uricase, when there was the transportation of uric acid from compartment C into compartment B. According to those definitions, the initial rate for the increase of valid SQ1_i_ or SQ2_i_ was an equivalent of the maximum SQ_i_, and thus depended on uricase dose when there was the rapid equilibrium among fast-diffusing uric acid in compartment A and B. Clearly, the limitation on transmembrane diffusion rate of uric acid into the dialysate before uricase action supported the saturable pharmacological action of the delivered uricase; the total quantity of multiple forms of diffusible uric acid was a determinant of the maximum of the pharmacodynamics for the removal of blood uric acid.

The removal of slow-diffusing uric acid in compartment C with hyperuricemic animal models by the delivered uricase was a marvelous advantage, and was detected by the following approach. The initial total quantity of fast-diffusing uric acid in plasma (TQ) was derived from its concentration before enzyme action and the sum of the total blood capacity plus 10 ml dialysis solution from the closed dialysate circuit (Supplementary Data S1 and Data S2). TQ thus considered only fast-diffusing uric acid pre-existed in blood and the dialysate before the action of uricase. In theory, after both the activity and action period of the delivered uricase were optimized to exhaust fast-diffusing uric acid originally found in the plasma before the action of the delivered uricase, the maximum SQ1_i_ was limited by TQ when slow-diffusing uric acid in compartment C was not involved, but higher than TQ when slow-diffusing uric acid in compartment C was involved. To detect the removal of slow-diffusing uric acid in compartment C, thus, the ratio of the maximum SQ1_i_ under such optimized conditions to TQ served as the index for comparison against a cutoff developed with reference system bearing no slow-diffusing uric acid and thus no transportation of uric acid among tissues. There was negligible slow-diffusing uric acid in blood *in vitro* when there were no precipitates of uric acid, since uric acid inside blood cells was negligible. Blood *in vitro* with uric acid level below the sum of its solubility limit and albumin binding capacity was suitable reference blood. Ovalbumin in goose blood is about 0.40 mM^14^; the dissociation constant of uric acid from albumin is about 20 μM^15^. Goose blood *in vitro* with 0.40 to 0.80 mM uric acid thus served as reference blood to develop the cutoff for comparison.

### Pharmacological efficacy *in vitro* of the delivered uricase

The hyperuricemia model *in vitro* with fresh goose blood bearing 0.40 to 0.80 mM uric acid was tested at first. During continuous running of the device minus uricase, uric acid concentrations in the two circuits of the delivery device reached equilibrium in 30 min and remained consistent in 6.0 h (Supplementary Data S3). After 6.0 h since vein injection, less than 20% activity of the native uricase was reserved in blood *in vivo*^16^,^17^,^18^. In the closed dialysate circuit, activities of the native uricase remained consistent in 6.0 h (Supplementary Data S3), supporting robust pharmacokinetics of the uricase *via* extracorporeal delivery.

**Figure 2:**
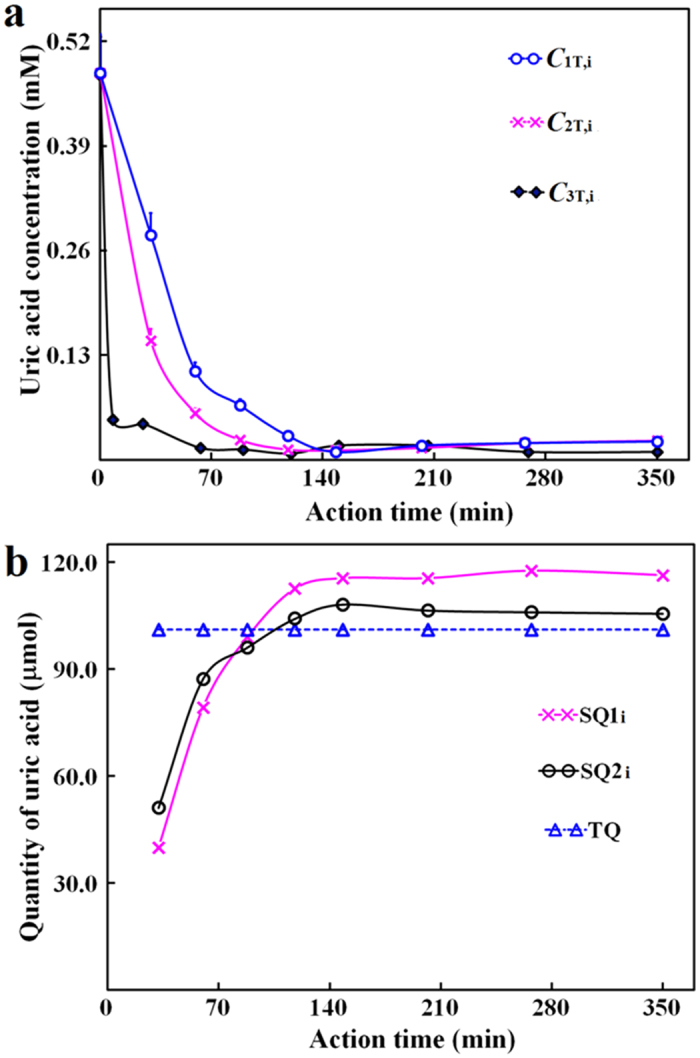
Changes of uric acid in hyperuricemia blood model *in vitro* and uricase pharmacodynamics. *C*_1T,i_: the concentration of uric acid in blood right before the dialyzer; *C*_2T,i_: the concentration of uric acid in blood right after the dialyzer; *C*_3T,i_: the concentration of uric acid in the closed dialysate circuit. Data processing details were provided in Supplementary Note S1. (**a**) *C*_1T,i_, *C*_2T,i_ and *C*_3T,i_ at 0.75 U uricase; (**b**) SQ1_i_, SQ2_i_ and TQ from data in (**a**).

At 0.30 U uricase, the three concentrations of uric acid continuously decreased in 3.3 h, with both faster rates and larger differences among them at the beginning stage, while slower rates at the later stage till their consistent bottoms below the quantification limit (Fig. 2a). Interestingly, SQ1_i_ continuously increased in a saturable manner till its plateau as the maximum, and correlated well with SQ2_i_ (Fig. 2b). Notably, the initial rate for the increase of SQ1_i_ or SQ2_i_ at 0.30 U uricase was smaller than that at 0.75 U uricase (Table 1; Supplementary Data S1 and Data S4), supporting the dose-dependent pharmacological action of the delivered uricase due to the rapid equilibrium of fast-diffusing uric acid in compartment A and B besides the validity of both SQ1_i_ and SQ2_i_. Hence, the delivered uricase was pharmacologically effective to remove uric acid in hyperuricemic blood *in vitro*, as reflected by the dynamic changes of both SQ1_i_ and SQ2_i_.

### Pharmacological efficacy *in vivo* of the delivered uricase with geese as model

Continuous running of the device minus uricase led to the equilibrium of uric acid in the two circuits after 50 min and consistent uric acid levels in 6.0 h (Supplementary Data S5), supporting the advantage to employ goose as hyperuricemia model *in vivo* since the control experiments were rather simple. However, there was about 30% decrease of uricase activity after continuous running of the device for 6.0 h (Supplementary Data S5); such a decrease was much smaller than that of the native uricase after circulation for 6.0 h *in vivo* through vein injection^14^,^15^,^16^, and may be due to the inactivation by reactive substances originated from the interactions of transitional metal ions with the excessive product hydrogen peroxide^19^.

**Figure 3:**
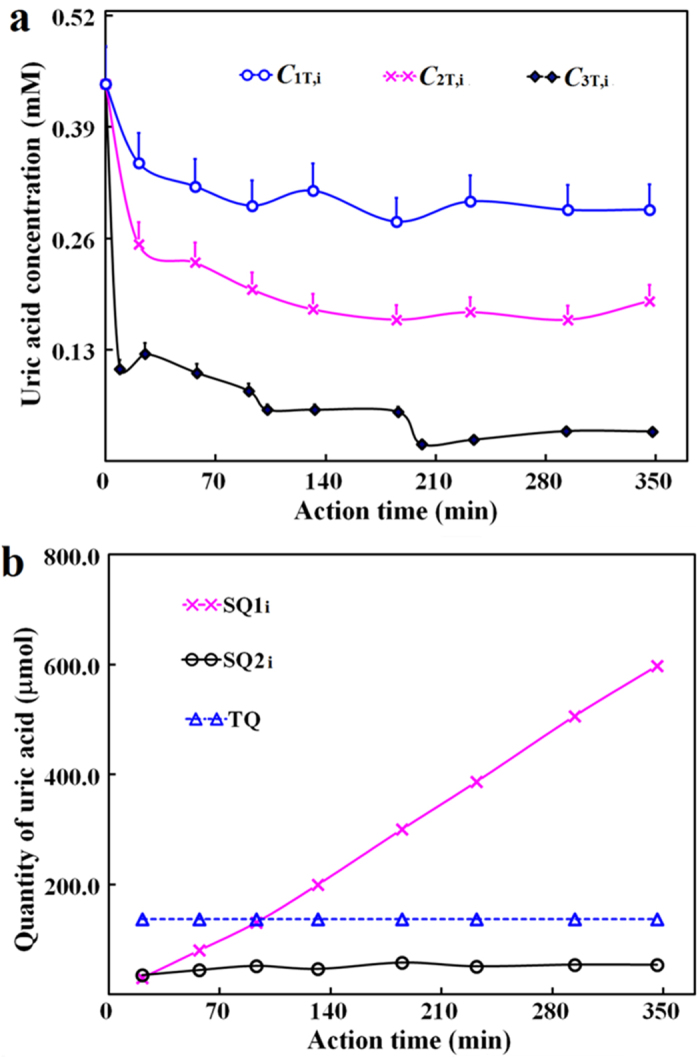
Changes of uric acid with goose as the hyperuricemia model *in vivo* and uricase pharmacodynamics. See legends of Fig. 2 for definitions of symbols and parameters. (**a**) *C*_1T,i_, *C*_2T,i_ and *C*_3T,i_ at 0.75 U uricase; (**b**) SQ1_i_, SQ2_i_ and TQ from data in (**a**).

With 0.75 U uricase, uric acid concentrations in blood before the dialyzer showed weaker decreases than those with blood *in vitro*, but the difference in uric acid levels right before and after the dialyzer was much larger than the equivalent with blood *in vitro* (Figs 2a and 3a). Unexpectedly, there were always negligible changes of SQ2_i_ *versus* action periods of the delivered uricase to support the invalidity of SQ2_i_ as the pharmacodynamics index; sometimes, there were even the increases of uric acid levels in blood *versus* action periods of the delivered uricase to qualitatively support the invalidity of SQ2_i_ as the index of pharmacodynamics (Fig. 3a,b; Supplementary Data S2). As expected, however, SQ1_i_ always continuously and almost linearly increased in 6.0 h, regardless of the change patterns of blood uric acid levels (Fig. 3b). After the action of the delivered uricase for 3.3 or 6.0 h, the maximum SQ1_i_ with blood *in vivo* still showed no association with uricase doses (Supplementary Data S4). However, the initial increase rate of SQ1_i_ at 1.5 U uricase was higher than that at 0.75 uricase, but consistent with that at 2.3 U uricase (Table 1), supporting dose-dependent and saturable pharmacological action of the delivered uricase. Therefore, the delivered uricase was effective to remove blood uric acid with hyperuricemia model *in vivo*, as reflected by the dynamic change of SQ1_i_, rather than that of SQ2_i_.

### Removal of multiple forms of uric acid *in vivo*

From the data with hyperuricemic blood *in vitro* bearing 0.40 to 0.80 mM uric acid, the ratio of the maximum SQ1_i_ for the exhaustion of fast-diffusing uric acid in blood to TQ was (0.96 ± 0.15) (*n* = 11), supporting negligible slow-diffusing uric acid in samples (Fig. 2b). At 99% confidence limit, the cutoff of the ratio of the maximum SQ1_i_ to TQ was about 1.37 with reference blood; the total blood capacity may reach 8% of body weight, giving a cutoff of 1.66 at 99% confidence limit to recognize the removal of slow-diffusing uric acid in compartment C with hyperuricemic geese (Supplementary Data S4).

With goose as hyperuricemic model *in vivo*, uricase doses were no less than 0.75 U and the action period was 6.0 h to facilitate exhausting fast-diffusing uric acid originally found in blood before uricase action (Table 1). In this case, the ratio of the maximum SQ1_i_ to TQ was (2.5 ± 0.7) (*n* = 15) after uricase action for 3.3 h, and (4.5 ± 1.3) (*n* = 15) after uricase action for 6.0 h. With data after uricase action for 3.3 h, the use of the cutoff of 1.37 found 14 samples whose slow-diffusing uric acid in compartment C was removed while the use of the cutoff of 1.66 found 13 samples involving the removal of slow-diffusing uric acid. With data after uricase action for 6.0 h, the use of the cutoff of 1.66 still found all the samples involving the removal of slow-diffusing uric acid. Therefore, the delivered uricase favorably and effectively removed multiple forms of uric acid with hyperuricemia model *in vivo*, as reflected by the ratio of the maximum SQ1_i_ to TQ after enzyme action for a sufficient period.

## Discussion

Through the extracorporeal delivery approach, the applicability of a native enzyme with an ideal action period since every delivery, and no immunoresponse even after repeated administration at proper intervals for a long time, are the greatest advantages. To remove the unwanted circulating small biochemicals as substrates with enzymes, therapeutic efficacy and safety are the primary concerns. Therapeutic efficacy of an enzyme requires its bioavailability, accessibility to the small substrate in blood and a long action period since every delivery. The conventional delivery approach employs vein infusion or injection through certain tissue to drive the enzyme to where the small substrate localizes, but the extracorporeal delivery approach utilizes semi-permeable dialyzer to selectively drive the small substrate to where the therapeutic enzyme localizes as long as the dialyzer has sufficient permeability. Both delivery approaches thus provide the required bioavailability and accessibility of the enzyme to its small substrate. Therapeutic safety requires nontoxic enzyme product(s), negligible removal of small nutrients/hormones, and negligible immunoresponse to the enzyme after repeated administration at proper intervals for a long time. The toxicity of product(s) and the removal of small nutrients/hormones by nonselective enzyme action(s) are the same with either delivery approach. Through conventional delivery, the problem of immunoresponse to an enzyme after repeated administration at proper intervals for a long time prevents its repeated use to treat a chronic disease over a long time. For any forms of an enzyme including a native one, the enzyme *via* extracorporeal delivery is not expected to elicit immunoresponse at all after repeated administration at proper intervals for a long time and can be expected to have a much longer period of action since every delivery. The closed dialysate circuit to circulate a small volume of dialysis solution of the enzyme may also prevent the dialysis disequilbrium syndrome and the imbalance of small nutrients/hormones. As a result, a native uricase, which cannot be repeatedly administrated at proper intervals for a long time *via* conventional delivery, may be readily administrated *via* extracorporeal delivery at proper intervals for continued treatment of refractory gout over a long time. However, more efforts are needed to prove the effectiveness of extracorporeal delivery to avoid the immunoresponse to a therapeutic enzyme after repeated administration at proper intervals for a long time, since hemodialysis was difficult to be administrated repeatedly to goose as the model *in vivo*.

The extracorporeal delivery approach may also be used to deliver macromolecular nonenzyme agents bearing selective interactions with unwanted small biochemicals in blood to release indiffusible or nontoxic products, such as reactive polymer scavengers that can selectively chelate or scavenge unwanted small biochemicals in the closed dialysate circuit. The extracorporeal delivery of such macromolecular nonenzyme agents is absorbing when no suitable enzymes are available, and can make artificial kidneys more favorable because of no burden on kidney excretion of enzyme products. Moreover, with a group of enzymes and/or macromolecular nonenzyme agents, multiple unwanted circulating small biochemicals can be removed concomitantly, and/or an unwanted small substrate can be converted into nontoxic product(s) *via* consecutive reactions, to advance artificial livers. Notably, as for the effective treatment of refractory gout, the use of high activity of uricase in the closed dialysate will produce a large quantity of hydrogen peroxide that will easily diffuse into blood. The toxicity of uricase product hydrogen peroxide is a problem for the treatment of refractory gout with uricase, which is more pronounced with patients bearing glucose-6-phosphate dehydrogenase deficiency. However, clearly, catalase whose activity is in great excess to that of uricase can be co-administrated in the closed dialysate to eliminate hydrogen peroxide, and meanwhile to regenerate free oxygen molecule in the closed dialysate to promote the action of the delivered uricase. The pharmacological efficacy and safety for the removal of blood uric acid by the co-administration of uricase and catalase through extracorporeal delivery are incomparable advantages over any conventional delivery. Furthermore, the device for extracorporeal delivery of therapeutic enzymes and macromolecular nonenzyme agents can be miniaturized to develop novel portable artificial kidney/livers for their uninterrupted long-term actions when such therapeutic macromolecular agents are supplemented at proper intervals, but molecular-adsorbent-recirculating-system cannot be miniaturized due to the consumption of dialysis solution^20^,^21^. Therefore, the extracorporeal delivery approach is promising for effective delivery of therapeutic enzymes and macromolecular nonenzyme agents to remove unwanted circulating small biochemicals causing/deteriorating chronic diseases.

## Methods

Geese were purchased from Guang’an Langde Goose Co. Ltd. (Guang’an, Sichuan, China, http://www.qy6.com/qyml/compuser8231.html), and braised in the Laboratory Animal Center of Chongqing Medical University under sanitary conditions. All experimental operations with geese were as gentle as possible to minimize suffering; all personnel involved in experimental operation with geese got the licenses from the Laboratory Animal Center of Chongqing Medical University. After treatment with the delivered uricase, geese were further braised under the same conditions after careful handling of wounds/cuts. This work was approved by the Ethical Commission of Chongqing Medical University; all the experimental methods involving geese were in accordance with the guidelines of the Animal Care and Use Committee of Chongqing Medical University; all experimental protocols involving geese were approved by the Animal Care and Use Committee of Chongqing Medical University.

Recombinant expression of the bacterial uricase was completely the same as reported previously^11^,^12^,^16^,^17^,^18^. Uricase activity was determined with 75 μM uric acid in sodium borate buffer at pH 7.4 by absorbance at 293 nm; one unit oxidizes one micromole substrate per min^14^,^15^,^16^. Blood was centrifuged at 3000 g for 10 min to get plasma. Each dialysate sample was immediately acidified with final 5% HClO_4_, and then neutralized with saturated K_2_HPO_4_ to get supernatant. Uric acid in plasma or supernatant of the dialysate was determined by a direct kinetic uricase method resistant to both hydrogen peroxide and reversible uricase inhibitors^22^. Other details of analysis were provided in Supplementary Note S2.

The dialysis buffer at pH ~7.3 was 10 mM sodium phosphate and 0.9% NaCl plus 27.0 g/L BSA (BSA sequesters free molecule of uric acid to facilitate its diffusion). The blood circuit in the dialyzer plus the connecting tubes has a capacity of about 28 ml and the capacity of the closed dialysate circuit was about 20 ml, but the capacity of the closed dialysate circuit was uncontrollable due to free diffusion of water and small solutes across the dialyzer membranes. Sodium heparin was used to prevent coagulation of blood. For the normal physiological states of geese in 8 h (no signs of salivation, lacrimation, tremors and gasping), the blood circuit was run at no more than 15 ml/min while the closed dialysate circuit was run at 20 ml/min to make homogenous solution. After the two circuits of the delivery device was run for an indicated time to achieve the equilibrium of uric acid, the native uricase in no more than 1.5 ml sterile dialysis buffer was added directly into the closed dialysate circuit.

For the removal of uric acid with hyperuricemia models *in vitro*, 200 ml fresh blood from one goose was placed in a glass of 500 ml to serve as the blood reservoir, which was magnetically stirred at 37 °C in water-bath in an isolated small room air-conditioned at 25 °C. The blood circuit was connected to the blood reservoir while 30 ml sterile dialysis solution was added to the closed dialysate circuit. All hyperuricemic blood models *in vitro* had uric acid from 0.40 to 0.80 mM. Uric acid reached equilibrium in the two circuits after running of the delivery device alone for 20.0 min (Supplementary Data S3). After continuous running of the delivery device alone for 30 min, 0.30 U uricase, 0.75 U uricase, or no uricase, in 1.50 ml sterile dialysis buffer was added directly into the closed dialysate circuit. After further running of the device for an indicated period since the addition of uricase, blood of 0.8 ml before and after the dialyzer, and the dialysate of 0.20 ml from the closed dialysate circuit, were sampled with a special order for analyses (Supplementary Note S1 and Note S2).

Geese of body weight over 5.0 kg and blood uric acid from 0.40 to 0.80 mM served as hyperuricemia models *in vivo*. Experiments were performed in an isolated small room air-conditioned at 25 °C and geese were kept in the room for more than 2 h before operation. The blood circuit was connected to goose artery and vein according to standard procedure of hemodialysis. The wings and legs of goose were tied with wide bandages to a frame to restrain its activity but provide the maximum comfort. After running of the delivery device alone for 50~60 min, no uricase as the control, the native uricase of 0.15 U per kg goose body weight, the native uricase of 0.30 U per kg goose body weight, or the native uricase of 0.45 U per kg, in 1.50 ml sterile dialysis buffer, was added into the closed dialysate circuit. After further running of the delivery device plus the delivered uricase for an indicated period, blood of 0.80 ml before and after the dialyzer and the dialysate of 0.20 ml were sampled with a special order for processing and analyses (Supplementary Note S1 and Note S2).

## Additional Information

**How to cite this article**: Zhang, C. *et al*. Extracorporeal delivery of a therapeutic enzyme. *Sci. Rep.* **6**, 30888; doi: 10.1038/srep30888 (2016).

## Supplementary Material

Supplementary Information

Supplementary Data S1

Supplementary Data S2

Supplementary Data S3

Supplementary Data S4

Supplementary Data S5

## Figures and Tables

**Figure 1:**
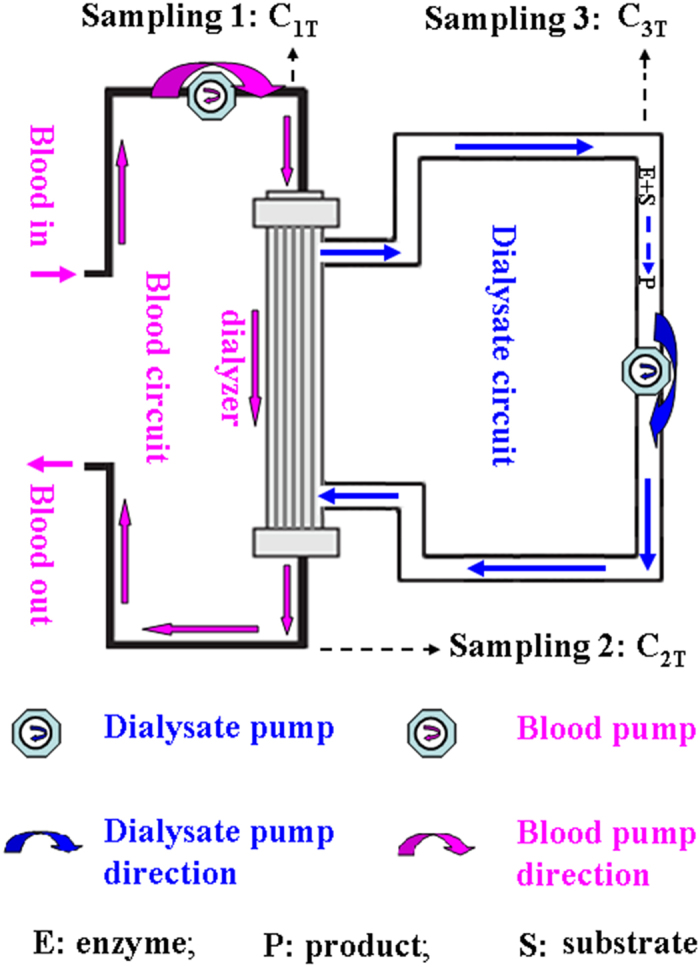
Work principle of the extracorporeal delivery device.

**Table 1:** Comparison of pharmacological action of the delivered uricase with hyperuricemic blood models *in vitro* and *in vivo*.

System	Uricase dose (U)	TQ^a^ (μmole)	Ratio of maxSQ1_i_ to TQ (3.3 h)	Ratio of maxSQ1_i_ to TQ (6.0 h)	Initial rate for SQ1_i_ (μmole/min)
*In vitro*	0.30 (6)	103 ± 19	0.88 ± 0.16	ND	0.78 ± 0.12
*In vitro*	0.75 (5)	155 ± 40	1.03 ± 0.18	ND	1.4 ± 0.3^b^
*In vivo*	0.75 (5)	136 ± 23	2.2 ± 0.7^c^	3.8 ± 1.2^c^	1.4 ± 0.4^d^
*In vivo*	1.5 (5)	203 ± 50	2.6 ± 0.9^c^	4.5 ± 1.6^c^	2.4 ± 0.8^e^
*In vivo*	2.3 (5)	153 ± 27	2.8 ± 0.3^c^	5.0 ± 0.6^c^	2.2 ± 0.5^e^

ND: not determined; maxSQ1_i_: the maximum SQ1_i_ after uricase action for the indicated period.

^a^indicates the total blood capacity of 210 ml with hyperuricemia models *in vitro* and of 310 ml with models *in vivo*;

^b^indicates *P* < 0.005 *versus* that with 0.3 U uricase *in vitro*;

^c^indicates *P* < 0.001 *versus* all the data *in vitro* including those with 0.3 and 0.75 U uricase after the action for 3.3 h;

^d^indicates *P* < 0.02 *versus* that with 0.30 U uricase *in vitro* while *P *> 0.9 *versus* that with 0.75 U uricase *in vitro*;

^e^indicates *P* < 0.05 *versus* that with 0.75 U uricase *in vivo*, and *versus* that with 0.75 U or 0.30 U uricase *in vitro.*
