# Supplementary material for: Extracorporeal delivery of a therapeutic enzyme
Source: Sci Rep. 2016 Aug 1;6:30888. doi: 10.1038/srep30888 (PMC4967896; doi:10.1038/srep30888)
Supplement: Supplementary Information [file srep30888-s1.doc]

**Supplementary Information to**

**Extracorporeal delivery of a therapeutic enzyme**

Chun Zhang^1,a^, Jun Pu^2,a^, Xiaolan Yang^1,a^, Tao Feng^1^, Fang Liu^1^, Deqiang Wang^1^, Xiaolei Hu^1^, Ang Gao^1^, Hongbo Liu^1^, Chang-Guo Zhan^3,*^, Fei Liao^1,*^

*^1^Key Laboratory of Medical Laboratory Diagnostics of the Education Ministry, College of Laboratory Medicine, Chongqing Medical University, No.1, Yixueyuan Road, Chongqing 400016, China;*

*^2^Department of Urology, the First Affiliated Hospital of Chongqing Medical University, No.1, Youyi Road, Chongqing 400016, China;*

*^3^Molecular Modeling and Biopharmaceutical Center and Department of Pharmaceutical Sciences, College of Pharmacy, University of Kentucky, 789 South Limestone Street, Lexington, KY 40536, U.S.A.*

___________________________________________________________________________

^a^ These authors contributed equally to this work.

^*^Corresponding authors.

Tel: +86-23-68485240;

Emails:

Zhan C-G, zhan@uky.edu,

Liao F, liaofeish@yeah.net

**Note S1.**

***Quantitative index of the pharmacological action of the delivered enzyme***

The quantity of uric acid eliminated within a short sampling interval during the action of the uricase delivered in the device (SQ_i_) reflects the instantaneous pharmacological action, and the sum of SQ_i_ over action periods started from the same initial timepoint for sampling as SQn_i_ index the pharmacodynamics for enzymatic removal of blood uric acid. During continuous running of two circuits of the delivery device for an indicated period, the instantaneous quantity of the small substrate eliminated within a short interval (QSE_i_) represented its instantaneous pharmacological efficacy. The sum of QSE_i_ by integration over the indicated period for continuous running of two circuits of the delivery device gave SQn_i_ as the accumulative pharmacological efficacy (the subscript i denoted the action period for continuous running of the two circuits of the delivery device). Clearly, shorter intervals were needed for reliable QSE_i_. In practice, QSE_i_ was calculated as the instantaneous quantity of the small substrate diffused from the blood circuit into the closed dialysate circuit within the interval (QST_i_) minus the instantaneous quantity of the small substrate deposited in the closed dialysate circuit within the same interval (QSD_i_), as described by Equ.(1); numerical integration of Equ.(1) thus gave SQn_i_ by Equ.(2). SQn_i_ reflected quantitatively the dynamic pharmacological action of the delivered enzyme or its pharmacodynamics.

To estimate the integrals of QST_i_ and QSD_i_, the concentration of fast-diffusing uric acid in blood right before the dialyzer (*C*_1T,i_), the concentration of fast-diffusing uric acid in blood right after the dialyzer (*C*_2T,i_) and the concentration of fast-diffusing uric acid in the dialysate (*C*_3T,i_) were determined by a special order (**Fig. 1**). The lag time for taking the two blood sample was denoted *△l*, which was the duration for a tiny volume of blood to pass exactly through the dialyzer and the interval for calculating QSE_i_. Blood capacity of the dialyzer was 20 mL and the flow speed of blood was fixed at 15 mL/min; *△l* was preset at 1.5 min for taking the two blood samples. In practice, after continuous running of the delivery device for an indicated period, an aliquot of blood before the dialyzer was taken to determine *C*_1T,i_, then an aliquot of the dialysate was taken at about 0.5 min for immediate termination of enzyme action and subsequent assay of *C*_3T,i_; finally an aliquot of blood after the dialyzer was taken at 1.5 min to determine *C*_2T,i_. Thus, the integrals of QSD_i_ and QST_i_ were estimated below.

In theory, QSD_i_ was the difference in the concentrations of the small substrate in the dialysate sampled at the two moments for taking the two blood samples to determine *C*_1T,i_ and *C*_2T,i_, times the capacity of the closed dialysate (CCD). Within the interval of *△l*, however, *C*_3T,i_ may show too tiny a change to give a reliable QSD_i_. Alternatively, *C*_3T,i_ of just one dialysate sample taken within the *△l* was determined to approximate QSD_i_ by Equ.(3) Unfortunately, QSD_i_ itself was inaccessible. In practice, *C*_3T,0_ was determined before the addition of enzyme; when there was a negligible change of *C*_3T,i_ within the last *△l*, the integral of QSD_i_ was approximated by Equ.(4) (**Note SS1**). With a reasonable enzyme activity after a long enough action period, there can be a negligible change of *C*_3T,i_ within the last *△l* and thus the validity of Equ.(4). Moreover, CCD was about 10% of TCB, the approximated integral of QSD_i_ by Equ.(4) was suitable to estimate SQn_i_ for reasonable reliability.

There were two approaches to QST_i_. The first approach employed the integration of the instantaneous transmembrane diffusion rate of the small substrate over the interval of *△l* to give QST1_i_, which should be proportional to the difference between *C*_2T,i_ and *C*_1T,i_ since transmembrane diffusion is the only force to cause the decrease of *C*_2T,i_ from *C*_1T,I_, and ultimately gave SQ1_i_. The second approach utilized the reduction of the small substrate in the blood circuit over the action period to yield the integral of QST2_i_, which can be approximated as the decrease in *C*_1T,i_ from *C*_1T,0_ times the total blood capacity (TCB) and finally gave SQ2­_i_.

To obtain QST1_i_ as defined, the estimations of the transmembrane diffusion rate constant of the small substrate, its concentration gradients both across the dialyzer membranes and along the blood flow direction in the dialyzer were mandatory, but were rather challenging. Alternatively, QST1_i_ was obtained as the difference in *C*_2T,i_ and *C*_1T,i_ times the volume of blood passed through the dialyzer within the interval of *△l*, by taking the difference in *C*_2T,i_ from *C*_1T,i_ as a constant within the interval of *△l* (**Fig. 1**). In detail, it was assumed that a tiny volume of blood (*dV*) in the connecting tube before the dialyzer was taken to determine *C*_1T,i_ while *C*_2T,i_ was determined artificially with the same blood sample after it had passed through the dialyzer at a fixed flow speed (*fs*). In this case, QST1_i_ was estimated by Equ.(5) *via* the integration over the stated interval of *△l* with *C*_2T,i_ and *C*_1T,i_ as constants and *△V* as the quantity of blood passed through the dialyzer. The integration of QST1_i_ over the given action period gave as Equ.(6). The process of numerical integration of Equ.(6) with *C*_2T,i_ and *C*_1T,i_ can adopt any effective strategies. QSE1_i_ was thus derived from QST1_i_ plus QSD_i_ while SQ1_i_ was from QSE1_i_. This approximation way to QST1_i_ ***preferred shorter spaces in given action periods and was applicable to common blood samples***. However, *C*_2T,i_ larger than *C*_1T,i_ was a outlier and this pair was discarded from computation.

The approximation of the integral of QST2_i_ ***assumes that there was a negligible quantity of the slow-diffusing small substrate involved in blood***. Considering less than 10% of whole blood was kept in the dialyzer and the connecting tubes, *C*_1T,i_ represented the instantaneous concentration of the small substrate in whole blood. QST2_i_ was calculated as the decrease of *C*_1T,i_ within the interval of *△l* times TCB, as Equ.(7), Clearly, QST2_i_ itself was inaccessible, but its integration was approximated as Equ.(8). ***This approximation applied to any spaces in given action periods***. QSE2_i_ was from QST2_i_ plus QSD_i_ while SQ2_i_ was from QSE2_i_. With any blood involving the slow-diffusing small substrate, *C*_1T,i_ may display a negligible decrease to invalidate the approximation and give unreliable SQ2_i_.

SQ1_i_ and SQ2_i_ will display concomitant saturation changes when there was a negligible quantity of the slow-diffusing small substrate; their initial rates may produce the expected dose-dependence on enzyme activities. Moreover, SQ1_i_ will be consistent with SQ2_i_ and limited by the initial total quantity of the fast-diffusing small substrate in the blood before enzyme action (TQ), which was equal to *C*_1T,0_ times TCB, when there was a negligible quantity of the slow-diffusing small substrate involved in blood, but higher than TQ and SQ2_i_ when there was a significant quantity of the slow-diffusing small substrate in blood. Such differences supported the removal of the slow-diffusing small substrate from blood.

**Note SS1. Approximation of QSD_i_**

As defined, for each short interval to estimate the instantaneous QSD_i_, the instantaneous concentrations of the small substrate in the closed dialysate circuit should be determined at the beginning (*C*_3T1,i_) and terminating points (*C*_3T2,i_) of the interval (*△t*) for determining *C*_1T,i_ and *C*_2T,i_, respectively, to give two concentration series as *C*_3T1,i_ and *C*_3T2,i_ (Fig. S1).


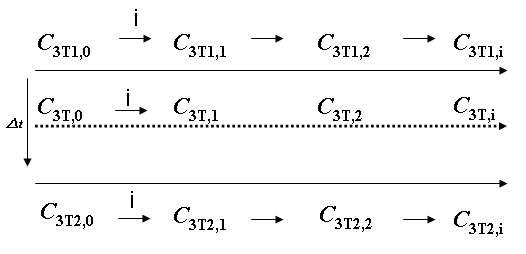


Fig S1. Two concentration series of the small substrate in the closed dialysate circuit.

To estimate SQ1_i_, the differences between i and i-1 in the time series of enzyme action can be the interval of *△l* for taking samples to determine *C*_1T,i_ and *C*_2T,i_. In this case, *C*_3T1,2_ will be consistent with C_3T2,1_, and *C*_3T1,i_ will be consistent with C_3T2,i-1_, and so on. As a result, . $C\text{3T1,0}$ was accessible by experimentation. When *C*_3T2,i_ was approximated by *C*_3T1,i_, there will be as long as there was a negligible difference in *C*_3T2,i_ and *C*_3T1,i_ in the i^th^ interval. At a fixed flow speed of blood, the change of *C*_3T,i_ in the i^th^ interval depended on enzyme activities in the dialysate as described below. With a low enzyme activity, there was usually a negligible change of *C*_3T,i_ in the i^th^ interval. With a high enzyme, there was a negligible *C*_3T,i_ after continuous running of the delivery device for a reasonable period and thus a negligible change of *C*_3T,i_ in the i^th^ interval. With a moderate enzyme activity, there was a negligible change of *C*_3T,i_ only when i was much larger. However, considering the total dialysate of 20 ml, the deviation was reasonable.

**Note S2. Additional experimental methods**

## Reagents, materials and chemicals

Uric acid and bovine serum albumin (BSA, fraction V) was from Sigma-Aldrich. Boric acid, Tri-(hydroxylmethyl)-aminomethane (Tris), isopropyl-β-D-thiogalactoside, sodium borate and other reagents were domestic reagents of analytical grade or better. Water was re-stilled before use. DEAE-cellulose was from Whatman (Kent, UK). All solutions were made sterile by filtration through 0.22 μm membranes.

## Recombinant expression, purification and activity assay of uricase

The expression of *Bacillus fastidious* uricase followed those described previously^11, 12, 16-18^. After induced expression at 16 ^0^C for 24 h, *Escherichia coli cells* (BL21, DE3) were harvested, and lysed by sonication treatment to yield a supernatant after centrifugation at 2000 × g for 20 min. Soluble uricase was purified over two consecutive DEAE-cellulose chromatography *via* the elution with 0.10 M Tris-HCl at pH 8.0 plus a linear gradient of NaCl from 0 to 0.40 M. Active fractions were pooled, concentrated to > 10.0 g/L with sucrose, dialyzed against the dialysis buffer (10 mM sodium phosphate plus 0.9% NaCl) for 48 h at 4 ^0^C with several changes of the buffer. Uricase activity was measured at 25 ^0^C with 75 μM uric acid in 50 mM sodium borate buffer at pH 7.4 by recording absorbance at 293 nm with Mapada UV-1600 PC spectrophotometer **^S1-S3^**. One unit of uricase oxidized one micromole of uric acid per min under stated conditions. The final preparation of the uricase bearing specific activity > 7.0 kU/g and the concentration > 7.0 g/L was filtered through 0.22 μm membrane and stored at 4 ^0^C before use in one week^11, 12^.

**Processing of samples for the quantification of uric acid and uricase inhibitors**

Blood sample was subjected to centrifugation at 2000 × g for 10 min to get plasma, in which uric acid was analyzed in duplicate. As for the dialysis solution, 10% solution of 60% perchloric acid was immediately added to terminate uricase action, and the mixture was rapidly neutralized and centrifuged to get the supernatants before analysis in duplicate.

To measure uricase inhibitors, a fixed activity of the uricase was used in the sodium borate buffer at pH 9.2 plus 25 μM uric acid to record reaction curve. *K*_m_/*V*_m_ estimated by a program with *K*_m_ preset at 0.30 mM was proportional to inhibitor equivalents**^S2-S6^**. To quantify uric acid, a direct kinetic uricase method resistant to inhibitors and H_2_O_2_ was used**^S5, S6^**. In 1145 μl solution of 50 mM sodium borate at pH 9.2, 50 μl sample was added, to measure the absorbance at 293 nm as the initial absorbance (*A*_0_) after the correction of dilution effects (0.4%); then, 5.0 μl solution of the recombinant uricase was added for final 40 U/L to monitor the change of absorbance at 293 nm and 25 ^0^C. With each sample, uricase reaction curve was recorded at 5-s intervals within 8.0 min, stored in a text file and read into computer memory for analysis to predict the absorbance after the completion of reaction (*A*_b_) **^S4^**. The difference between *A*_0_ and *A*_b_ was the net absorbance of uric acid. Concentration of uric acid were calculated with the millimolar absorptivity of 11.5 (mM)^-1^·cm^-1^ **^S6^**.

## Data processing and statistic analysis

The new quantitative index of the pharmacological action was derived in context. To estimate the initial rate for the change of the new quantitative indexes of pharmacological action, the first part of linear increase was analyzed by regression. The whole blood capacity of goose in ml was assumed to be 6% of the body weight in kg, unless otherwise stated. Results were presented as mean (*x*) ± standard deviation (SD), and compared by Student’s *t*-test in MS Excel 6.0 with *P* < 0.05 as the confidence limit for difference.

**Additional references**

1. Feng J, *et al*. Comparison of activity indexes for recognizing enzyme mutants of higher activity with uricase as model. *Chem. Cent. J.* **7**, 69 (2013).
2. Liu M, Yang X, Yuan Y, Tao J & Liao F. PCFenzyme for kinetic analyses of enzyme reaction processes. *Procedia Environm. Sci.* **8**, 582-587 (2011).
3. Liu B, *et al*. An integration strategy to estimate the initial rates of enzyme reactions with much expanded linear ranges using uricases as models. *Anal. Chim. Acta* **631**,22-28 (2009).
4. Liao F, *et al*. Evaluation of a kinetic uricase method for serum uric acid assay by predicting background absorbance of uricase reaction solution with an integrated method. *J. Zhejiang Univ. Sci. B.* **7**, 497-502 (2006).
5. Zhao Y, *et al*. Characterization of a uricase from Bacillus fastidious A.T.C.C. 26904 and its application to serum uric acid assay by a patented kinetic uricase method. *Biotechnol. Appl. Biochem.* **45**, 75-80 (2006).
6. Liao F, Zhu X, Wang Y & Zuo Y. The comparison of the estimation of enzyme kinetic parameters by fitting reaction curve to the integrated Michaelis-Menten rate equations of different predictor variables. *J. Biochem. Biophys. Methods.* **62**, 13-24 (2005).
